# Translational Research in Vitiligo

**DOI:** 10.3389/fimmu.2021.624517

**Published:** 2021-03-02

**Authors:** Erica L. Katz, John E. Harris

**Affiliations:** Department of Medicine, Division of Dermatology, University of Massachusetts Medical School, Worcester, MA, United States

**Keywords:** vitiligo, translational research, autoimmunity, melanocyte oxidative stress, genetics

## Abstract

Vitiligo is a disease of the skin characterized by the appearance of white spots. Significant progress has been made in understanding vitiligo pathogenesis over the past 30 years, but only through perseverance, collaboration, and open-minded discussion. Early hypotheses considered roles for innervation, microvascular anomalies, oxidative stress, defects in melanocyte adhesion, autoimmunity, somatic mosaicism, and genetics. Because theories about pathogenesis drive experimental design, focus, and even therapeutic approach, it is important to consider their impact on our current understanding about vitiligo. Animal models allow researchers to perform mechanistic studies, and the development of improved patient sample collection methods provides a platform for translational studies in vitiligo that can also be applied to understand other autoimmune diseases that are more difficult to study in human samples. Here we discuss the history of vitiligo translational research, recent advances, and their implications for new treatment approaches.

## Introduction: Early Research in Vitiligo

**Figure 1:**
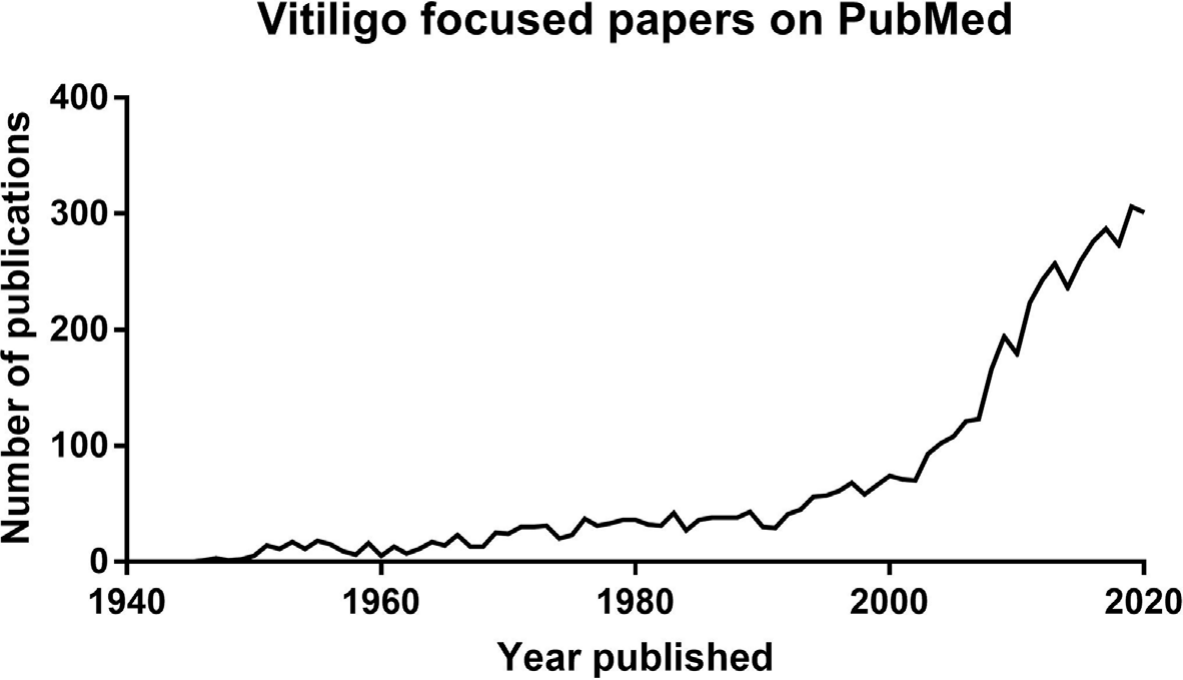
Graph showing the exponential increase of publications available on Pubmed with titles including vitiligo published over time.

Focused research in vitiligo has surged over the past 30 years. This is revealed by the exponential increase in scientific papers published with vitiligo in the title (**Figure 1**) and significant progress in developing new targeted treatment approaches. However, the first documentation of vitiligo and its treatment exists from 3,500 years ago. These ancient texts describe the appearance of vitiligo, emphasize the social stigma associated with the disease, and list treatment approaches used at the time. Vitiligo patients still experience the social stigmas documented in ancient Indian and Buddhist writings, including the restriction of marriage (1). Most of the treatments noted in these ancient texts, including the application of elephant stool or ingestion of cobra bones, are not currently utilized. However, the basis of one remedy is still used today. According to the Atharvaveda, an ancient Indian medical text written in ~1400 B.C., patients with vitiligo were instructed to chew on black seeds from the Bavachi (*Psoralea corylifolia*) plant, which was later determined to be a source of psoralen. They then sat in the afternoon sun until they began to sweat profusely, which led to blistering sunburns of their exposed skin. While this treatment is unacceptable by today’s standards, it would have likely been effective, as evidenced by its continued use in ancient times and its later rediscovery in the mid 1900’s as topical or oral psoralen derivatives in combination with UVA light treatment, currently known as (PUVA) (2).

Vitiligo has been discussed in modern medical literature for over one hundred years. These early records were primarily case studies characterizing clinical observations and their correlation with other diseases. Vitiligo was described as white patchy depigmentation of the skin beginning as small macules that gradually increased and coalesced into larger patches over time, frequently sparing the hair pigment (3). Authors noted that patients with vitiligo exhibited a higher incidence of other autoimmune diseases, such as alopecia areata, type 1 diabetes, and Addison’s Disease (4). The documented therapeutic approaches for treating vitiligo included arsenic, sulfur, mercury, antimony, and sulphuric acid, which are thankfully not used any longer due to limited efficacy and the potential for severe side effects (5). Sulfuric acid was used to burn the skin at borders of the lesions to make them less distinct, and therefore less obvious to the observer. The tolerance of these toxic approaches highlights the motivation of patients to treat their disease.

It was not until the 1940s when more in-depth research studies were performed. Haxthausen in 1947 reported that grafting vitiligo lesional skin onto non-lesional sites led to varying degrees of repigmentation of the lesional skin, while transplanting nonlesional skin onto a lesional area led to the graft depigmentation. He hypothesized that the local environment, which might be determined by the nervous system, influenced the pigmentation state of the graft (6). Comel, in 1948, reported similar results with pedicled and tubed flaps. The lesional flaps repigmented, while the nonlesional flaps attached to lesional skin depigmented (7). Conversely, Spencer and Tolmach in 1951 reported that graft transplantation between lesional and nonlesional skin did not lead to the repigmentation of lesional grafts or depigmentation of nonlesional grafts, but reported extension of depigmentation around the lesional skin graft (8). A possible reason for the conflicting results is the different methodologies used for the skin grafts. Haxthausen used thin split thickness grafts, referred to as a Thiersch graft, while Spencer and Tolmach used full thickness skin grafts. Although these were small case studies that showed mixed results, they suggested that the environment in which the graft is transplanted is an important factor. It wasn’t until 1964 when Behl applied skin grafts as a large scale treatment option for vitiligo (9), utilizing the Thiersch grafting technique similar to the Haxthausen study and limiting the variability in repigmentation and relapse by restricting the procedure to only those with stable disease.

## Pathogenesis of Vitiligo

**Figure 2:**
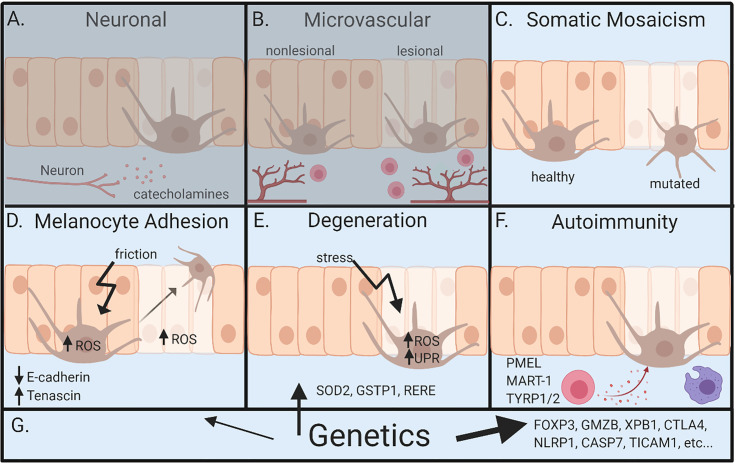
Overview of pathogenesis for vitiligo. **(A)** Neuronal involvement – neurons within the skin release neuropeptides like catecholamines, which act on melanocytes and lead to depigmentation. **(B)** Microvascular theory – vitiligo lesions have increased blood flow during segmental vitiligo, which allows for increased infiltration of lymphocytes that results in autoimmune attack of melanocytes. **(C)** Somatic mosaicism – depigmentation develops because a somatic mutation in melanocytes leads to genetically distinct populations that are susceptible to autoimmune attack. **(D)** Melanocyte adhesion – friction or oxidative stress in melanocytes or keratinocytes leads to melanocyte loss because of reduced adhesion to the skin. **(E)** Degenerative theory – depigmentation occurs because of intrinsic melanocyte defects, such as increased susceptibility to environmental stressors and dysregulation of reactive oxidative species (ROS). **(F)** Autoimmunity theory – autoreactive immune cells attack and kill melanocytes, ultimately leading to depigmentation. **(G)** Genetics – underlies all pathways leading to vitiligo. Genetic studies most clearly implicate autoimmunity, but also melanocyte contributions. Neuronal and microvascular theories are least supported, represented by **(A, B)** being grayed. Figure created in BioRender.com.

There are a number of hypotheses offered to address the pathogenesis of vitiligo. These hypotheses consider roles for innervation, microvascular anomalies, melanocyte degeneration from oxidative stress, defects in melanocyte adhesion, autoimmunity, somatic mosaicism, and genetic influences (1, 10–15) (**Figures 2A–G**). Some have strong evidence to support them, while others are relatively unsupported but continue to be discussed. We will outline these hypotheses and the evidence offered to support them below.

### Neural Theory

The hypothesis that innervation influences depigmentation, often labeled the “neural hypothesis”, is primarily based on a few largely unsupported observations: 1) Segmental vitiligo is unilateral, and therefore mistakenly labeled as dermatomal; however, disease is rarely limited to a single dermatome, and often crosses many dermatomes (11, 16–18). 2) Lerner hypothesized that vitiligo is caused by the increase of neuropeptides released by neurons and that this decreases melanocyte melanin production (19). Catecholamines have also been reported to be elevated in the urine of vitiligo patients, leading some to assume this came from dysfunctional neurons (20–22). However, others dispute these results and note catecholamines are also produced by melanocytes (23, 24). Thus, these studies do not implicate nerves in disease pathogenesis. 3) There are case reports of vitiligo preferentially affecting limbs that had been traumatically denervated, but the opposite is also true, that denervated limbs have been reported to repigment or be spared from vitiligo altogether (19, 25, 26). In most cases, denervation does not affect vitiligo. Thus, these discordant case reports are more likely chance observations than actual indications of disease pathogenesis. 4) Sweating and vasoconstriction have been reported to be altered in lesions (27, 28). However, many reports are contradictory. Some reports claim an increase in cholinergic influence in all vitiligo lesions, while others report this observation occurs in segmental lesions but not bilateral lesions. Data on the level of sympathetic tone is also conflicting, as increased sympathetic tone has been observed by some groups while others observed decreased sympathetic tone (27–34). Since these studies are largely inconclusive, it is premature to suggest they provide any insight into vitiligo pathogenesis. 5) Stress or trauma appears to exacerbate vitiligo (19). This is often thought to be due to neurohormonal mechanisms, however there is currently no evidence to support neuronal involvement. Many autoimmune conditions are exacerbated by stress, an observation that has not implicated neurons in their pathogenesis. 6) Animal studies reveal neural control of pigmentation, particularly in fish (33, 35). However, there is no evidence for this in mammals, especially humans (36). Thus, we suggest future discussions defer mention of the “neural hypothesis”, at least until there is solid evidence available to support it.

### Microvascular Theory

The microvascular theory suggests there is an increased blood flow into lesional skin during segmental vitiligo. The use of iontophoresis and laser Doppler flowmetry demonstrated up to triple the blood flow in lesional skin of segmental vitiligo patients compared to healthy, but not within nonsegmental lesional skin (37). The unilateral presentation was proposed to be from the activation and expansion of the autoreactive T cells in the regional lymph nodes that then exit into the blood circulation. An increased blood flow may then escalate the migration of lymphocytes, specifically melanocyte-specific cytotoxic T cells because of their expression of cutaneous homing receptors, to the lesion for the destruction of melanocytes (13, 38–40). However, a contradictory observation is that when vitiligo develops in melanoma patients as the result of a vaccine injected for the expansion of melanocyte-specific T cells, disease is typically reported to develop in a nonsegmental pattern. If the microvascular theory were true, then it would be expected to develop in a segmental pattern originating in the region of the injection site (12). Furthermore, the microvascular theory is often grouped closely with the neural theory because it has been proposed that damaged sympathetic nerves leads to the increased blood flow due to the vasodilation of the skin blood vessels (41). The lack of real evidence for this theory supports discarding it until further studies provide support.

### Degenerative Theory

The degenerative theory focused on the idea that there is an intrinsic defect within melanocytes that leads to their loss within the skin, thereby causing depigmentation. This theory arose from observations that melanocytes from vitiligo patients are difficult to culture and proliferate much slower than melanocytes from healthy individuals (42). Additionally, dysregulation of reactive oxidative species (ROS) and increased susceptibility to chemicals that induce ROS have been reported in melanocytes from patients with vitiligo (43, 44). Catalase is an enzyme that converts hydrogen peroxide, a source of ROS, into water, and expression of this enzyme is reportedly reduced in vitiligo lesional skin (45). Attempts to mitigate oxidative stress in melanocytes using a topical psuedocatalase cream does not seem to have an impact on disease, but use in combinational treatments with calcium and UVB light treatments have been reported to result in effective repigmentation (46, 47). However, Gilhar et al. demonstrated that melanocytes are functional within lesions because human vitiligo skin grafted onto nude mice fully repigmented and the number of melanocytes within the graft increased (48). Therefore, although there is clear evidence indicating melanocytes are abnormal in lesional skin, the idea that vitiligo exclusively develops from intrinsic melanocyte degeneration is unlikely because of the disputing studies supporting the fact that melanocyte defects are not sufficient to cause disease.

### Melanocyte Adhesion Theory

The melanocyte adhesion theory suggests that melanocytes lose or have decreased adhesion to the skin and are therefore easily eliminated or otherwise lost during oxidative stress or mechanical pressure, such as friction from clothing (49). Some studies report a decreased expression of the adhesion molecule E-cadherin on melanocytes, while another reported no significant differences in expression of adhesion molecules between healthy, nonlesional, and lesional skin but elevated expression of an “anti-adhesion molecule” tenascin in vitiligo lesional and nonlesional skin, which might lead to the loss of melanocytes within the skin (50, 51). More *in vivo* data is needed to determine the role of melanocyte adhesion defects during melanocyte loss, such as whether adhesion molecules knocked out in melanocytes in mouse models lead to increased disease severity. Keratinocytes may also play a role in the loss of melanocytes. Keratinocytes and melanocytes comprise a “epidermal-melanin unit” and maintain homeostasis in the epidermis through paracrine signaling of growth and survival factors (52). Studies report that keratinocytes within vitiligo perilesional and lesional skin have dysregulation of oxidative stress (52). For example, keratinocytes have mitochondrial abnormalities evident from increased ROS production, lower production of antioxidants, and expression of apoptotic proteins (53, 54). Keratinocytes clearly have a role in pathogenesis, as their production of CXCL9 and CXCL10 recruits autoreactive CD8+ T cells to the skin (55). Therefore, it is conceivable that intrinsic defects within keratinocytes may play contribute to abnormalities in melanocyte adhesion during vitiligo pathogenesis. The current available data is intriguing and may support a role in vitiligo pathogenesis. The loss of melanocyte adhesion to keratinocytes might be a cause or effect of autoimmune inflammation. For example, it may trigger autoimmunity by a large release of autoantigens, or intrinsic defects in handling stress or autoimmune attack itself might lead to reduced adhesion (56).

### Autoimmunity Theory

The autoimmunity theory offered that self-reactive immune cells attack melanocytes, which then leads to their death and loss from the skin, resulting in depigmentation. This was one of the earliest theories, since initial case studies noted the coincident onset of vitiligo with other autoimmune diseases, thus implicating autoimmunity by association. In the late 1970s, a study revealed the presence of autoantibodies against melanocytes only in vitiligo patients and not in healthy controls using immunofluorescence complement fixation tests (57). This supported the notion that autoimmunity played a functional role in disease because it indicated activation of adaptive immunity specifically against melanocytes. Later studies revealed that autoantibodies could lead to the destruction of melanocytes *in vivo* by grafting human skin onto nude mice and subsequently injecting purified IgG antibodies from vitiligo patients (58). This resulted in a decrease of melanocytes in the grafts on mice injected with vitiligo patient IgG compared to healthy patient control IgG (58). It was later determined that autoantibodies are likely not the main drivers of disease because autoantibody levels do not correlate with disease severity in vitiligo patients and autoantibodies are equally distributed throughout the body, rather than concentrated in lesions (59).

While autoantibodies indicated an autoimmune response against melanocytes in vitiligo, it became apparent that melanocyte damage results from another mechanism. Early immunohistochemical (IHC) studies noted the decrease of melanocytes within lesional skin and the presence of immune cells in the upper dermis and epidermis (33, 60). It was then reported that CD8+ T cells infiltrate the skin and are positioned within close proximity to dying melanocytes (39, 61). Van den Boorn et al. determined that the immune system, and specifically CD8+ T cells, were both necessary and sufficient for melanocyte destruction in the skin of vitiligo patients by incubating T cells isolated from perilesional skin with nonlesional skin explants from the same donor, which resulted in melanocyte death (62). Furthermore, autoimmunity is supported by genome-wide association (GWA) studies that revealed genes associated with vitiligo risk are predominately immune-related, including genes associated with both adaptive and innate immune responses (63).

Melanocytes during vitiligo appear to be abnormal at baseline, producing heat shock proteins to help manage cellular stress, which activate the unfolded protein stress response. These abnormalities may lead to the release of damage-associated molecular patterns (DAMPs), which signal through pattern recognition receptors (PRRs) like TLR2, TLR4, and NLRP inflammasomes (64). Vitiligo-inducing chemicals like phenols initiate the unfolded protein response (UPR) in melanocytes, which results in the production of IL-6 and IL-8 (65), two cytokines that promote immune cell recruitment. Additional studies report infiltration of vitiligo lesional skin by innate immune cells, including inflammatory dendritic cells, macrophages, and natural killer (NK) cells (39, 66, 67). Yu et al. reported an increase of NK cells in vitiligo nonlesional skin, suggesting a role in the onset of disease (67). These studies suggest that melanocytes may initiate the innate immune response through the recruitment of innate immune cells from inflammatory signals and the release of DAMPs that are able to signal through pathogen recognition receptors (PRRs) on these cells. Additional studies will be important to further support these observations and enhance our understanding how the innate immune system bridges melanocyte stress and adaptive autoimmunity in vitiligo.

### Genetics

Genetics clearly influences the risk of developing vitiligo, although should not be described as a theory in itself. Indeed, genetic variation influences all pathways in the body, including immunology, melanocyte stress and adhesion, etc. Several epidemiological and familial studies addressing the incidence of vitiligo report that vitiligo affects 1%–2% of the overall population and that there is a higher risk for people who have first-degree relatives (FDR) with disease (68–71). Many of these studies were limited due to the low number of participants, although a recent study by Kim et al. incorporated a large number of individuals in a Korean population, providing significant power in their analysis. Their study calculated incidence risk ratio (IRR) for FDRs and found that siblings of vitiligo patients have a higher incidence of disease, with a larger increase if the sibling is a twin (69, 71, 72). Unlike previous studies, Kim et al. also reported a higher IRR for offspring that have a mother with vitiligo compared to a father (71). Although this increased risk could be from shared environmental risks, another possibility is the inheritance of mitochondrial DNA that is only obtainable maternally. Overall, homogeneity of the study population provided strong, powered data about vitiligo heritability, but more studies will be needed to determine whether these observations can be applied to other ethnicities and mixed populations.

Recent GWA studies revealed small nucleotide polymorphisms (SNPs) in many immune-related and melanocyte specific genes (63). The identification of melanocyte-specific genes (OCA2-HERC2, PMEL) through GWAS support a role for melanocyte involvement, while genes involving stress and apoptosis (SOD2, GSTP1, RERE, XPB1) support a role for melanocyte stress as well (68, 73, 74). The autoimmunity theory is validated by GWA study hits, as well with the melanocyte-specific genes are often autoantigens (PMEL) and immune-related genes (FOXP3, GMZB, XPB1). While these SNPs have provided significant insight, functional genomic studies are needed to understand the full relationship SNPs have on disease pathogenesis. However, it is difficult to identify gene target of SNPs because most occur in noncoding regions that likely regulate gene transcription, which may be present at a significant distance from their target genes. Recent three-dimensional (3D) genome analysis studies have reported that SNPs can influence genes that are linearly distant because of higher-order chromatin structures allow interactions of distant genome regions (75–77).

While genetics clearly plays a role in vitiligo, environmental exposures, and stochastic events factor into pathogenesis as well. This is indicated by the fact that coincident rates for identical twins to develop vitiligo is only 23%–26%, rather than the expected 100% if pathogenesis was only influenced by genetics (69, 71). Chemicals in permanent hair dyes and cosmetics, as well as chemicals like 4-TBP and monobenyzl ether of hydroquinone, induce and exacerbate vitiligo (78–80). This is likely through the induction of cellular stress pathways within melanocytes when they attempt to convert the chemical into melanin, as the chemicals are very similar in structure to the amino acid tyrosine, the building block of melanin synthesis. Additionally, stochastic events within the immune system may result in the develop of vitiligo because T cell receptors and antibodies are generated through random recombination of existing genes. Thus, a single panel of genetic factors as found in identical twins could result in very different immune responses through stochastic events. Therefore, a combination of genetic predisposition, environmental exposures, and stochastic events contribute to the risk of developing vitiligo.

## Segmental Vitiligo

While vitiligo is typically bilateral and symmetric, segmental vitiligo is a subtype of the disease that presents as unilateral depigmentation that does not cross the midline. Historically, the segmental subtype has been misnamed “dermatomal vitiligo” because its unilateral presentation was reminiscent of shingles, which affects the skin in a distribution overlying a single sensory nerve, which also localizes unilaterally. However, segmental vitiligo rarely, if ever, follows a single dermatome, but frequently involves multiple dermatomes and even runs perpendicular to dermatomes, indicating that this disease is not related to single nerves (12). Because of its unilateral nature and dermatomal misnomer, many believed that segmental vitiligo may have a separate and distinct pathogenesis, hypothesizing that nerves might play a role in this subtype of disease. However, more recent studies have shed light on mechanisms that lead to segmental vitiligo, including strong evidence that it is also driven by autoimmunity (40, 62).

In fact, there may be an important role for somatic mosaicism in the pathogenesis of segmental vitiligo. Somatic mosaicism occurs when there are two genetically distinct populations of cells arising from a mutation that appears within the zygote during development. A post-zygotic mutation occurring in an embryonic melanocyte can be passed on to daughter cells that then migrate ventrally from the neural crest (81). The somatic mosaicism theory suggests that a somatic mutation creates susceptible melanocytes by inducing melanocyte stress or increasing the expression of melanocyte specific markers (12), which then become the targets for autoimmune attack that is limited to the mutant population of cells (82). The ventral migration of these melanocytes during development without crossing the midline may lead to the unilateral distribution of disease, characteristic of segmental vitiligo (11). Future studies will be important to test this hypothesis directly.

## Hypotheses Influence Research Questions and Data Interpretation

Hypotheses that address the pathogenesis of disease influence experimental design, as well as data interpretation, which can be either beneficial or detrimental to progress in understanding disease, as this natural bias can focus investigation leading to accelerated discovery, or negatively influence data interpretation within a narrow framework. This has been done in some respect with the “neural hypothesis”. As discussed above, the characteristic unilateral nature of segmental vitiligo for years was misinterpreted as supportive of neural involvement, despite the fact that lesions did not follow specific dermatome patterns at all (11). In addition, the presence of elevated catecholamines in the blood and urine of vitiligo patients were interpreted to indicate support for the neural hypothesis, suggesting that these catecholamines emanated from dysfunctional nerves (21). An alternative interpretation proposed that the increased catecholamines were a product of melanocyte destruction. This interpretation led to studies that observed that melanocytes also produce catecholamines, thus providing new insight about melanocyte biology during disease and further refutes the neural hypothesis (23, 24). Discussions and new interpretations when exploring both proposed hypotheses about the presence of catecholamines and nerve mapping helped drive focus to more promising pathogenesis ideas.

Another example highlighting different data interpretations driving experimental approaches is the observation that immune cells infiltrate vitiligo lesions. Immunohistochemical studies revealed infiltrating immune cells within the upper dermis and epidermis of vitiligo lesions (33, 60). It was then identified these immune infiltrates were predominantly CD8+ T cells and macrophages, which were positioned within close proximity to dying melanocytes (39, 61). Some interpreted these immune cells as simply “cleaning up” after melanocyte degeneration, which led to exploration of the role of macrophage clearance of debris in promoting repigmentation (83). Alternatively, researchers hypothesized immune infiltration into the epidermis supported a direct autoimmune attack against melanocytes. This led to additional characterization of the infiltrating T cells, which reported a higher frequency of melanocyte specific CD8+ T cells in the circulation of vitiligo patients expressing the skin homing receptor cutaneous leukocyte-associated antigen (CLA) (38, 84, 85). Further studies on T cells isolated directly from lesional skin revealed that cytotoxic CD8+ T cells selectively kill melanocytes, further validating the autoimmune theory (62).

In addition, increased production of heat shock proteins (HSPs) was observed in melanocytes from patients with vitiligo. One interpretation of this data in the context of the degenerative theory is that the elevated HSPs are simply a result of increased melanocyte stress from intrinsic defects in regulating ROS production. This led to the exploration of melanocyte ROS and HSP production during normal cellular processes and under environmental stressors (65, 86–88). Another interpretation is that the increased production of HSPs by melanocytes acts as danger signals that induce inflammation, leading to autoimmunity. With this in mind, studies indicate that HSP70i is elevated in human melanocytes during chemical-induced vitiligo, has the capacity to activate skin dendritic cells, and is required for the induction of vitiligo in mouse models (66, 89).

In fact, both possibilities may be true. Melanocyte intrinsic defects regulating ROS production may play in a role in activating autoimmunity. The interplay of endoplasmic reticulum (ER) stress and ROS production have been implicated in the development of multiple autoimmune diseases, including vitiligo (90–92). Melanocytes within vitiligo skin show signs of oxidative and ER stress, including elevated ROS, a dilated ER, as well as gene expression consistent with UPR activation, including X-box protein 1 (XBP1), a transcription factor that is needed to produce cytokines involved in innate immune cell recruitment (65, 87). Vitiligo-inducing phenols induce expression of XBP1 in melanocytes (93), providing a feasible connection between melanocyte oxidative and ER stress to the immune response. Furthermore, these stress pathways within the melanocytes lead to the production of HSPs to help mitigate this stress, which are then secreted from the melanocyte or released during cell death to signal through TLRs and other PRRs to activate the immune response. Exploring multiple hypotheses, such as melanocyte stress, innate immune activation, and adaptive autoimmunity through complementary approaches will give a better understanding of the complex cell interactions and mechanisms at play during vitiligo pathogenesis.

## Addressing Existing Hypotheses in Future Research

To further explore existing hypotheses and to better understand how they interface to cause vitiligo, genetic and functional analyses comparing lesional, nonlesional, and healthy skin will be needed. Tracing mutations within melanocyte lineages will interrogate somatic mosaicism development and progression. Performing single cell RNA sequencing and functional genomics techniques like Assay for Transposase-Accessible Chromatin using sequencing (ATAC-seq) can guide functional studies addressing the mutations found in lineage tracing to determine their direct role on melanocyte homeostasis and function.

The results of genetic studies have launched many experiments. Since many GWAS hits are in both melanocyte and immune related genes, the identified genomic risk alleles have guided studies that address both the degenerative and autoimmunity theories. Studies to dissect intrinsic pathways within melanocytes have provided insight about stress responses, ROS production, metabolism, and adhesion. These insights have led to new questions that have yet to be answered, such as understanding the interface between melanocytes and inflammation. For example, future studies may dissect how melanocyte stress and inflammation influence each other during disease initiation and progression. Stressed melanocytes may induce local inflammation that then initiates and perpetuates autoimmunity in vitiligo. Inflammation could be also a stressor to melanocytes, and since melanocytes are defective in vitiligo this stress may weaken the melanocytes further, exacerbating autoimmune attack.

Additionally, the interplay of inflammation and melanocyte adhesion should be pursued. Skin inflammation could be reducing melanocyte adhesion or melanocytes that lose adhesion could be triggering inflammation and breaking tolerance. One reason for these unanswered questions is that cell interactions are not as easily studied in cell culture because multiple cell types interact within the tissue within a 3-dimensional (3D) environment. 3D skin models and animal models could be useful tools for exploring these questions. These models will also be helpful in working to define the complex, multicellular interactions that characterize autoimmunity within the skin. Unanswered questions include the mechanism of autoimmune melanocyte killing, tolerance mechanisms for suppression or prevention of disease, and whether other immune cells besides autoreactive CD8+ T cells contribute to melanocyte death.

These questions will be elucidated by creatively utilizing developing technologies and tools. Interest in understanding these interactions has supported the advancement and utilization of translational research tools such as flow cytometry, cell sorting, and live *in vivo* imaging. As these technologies develop, opportunities to explore these cellular interactions will expand.

## Translational Tools for Vitiligo Research

**Figure 3:**
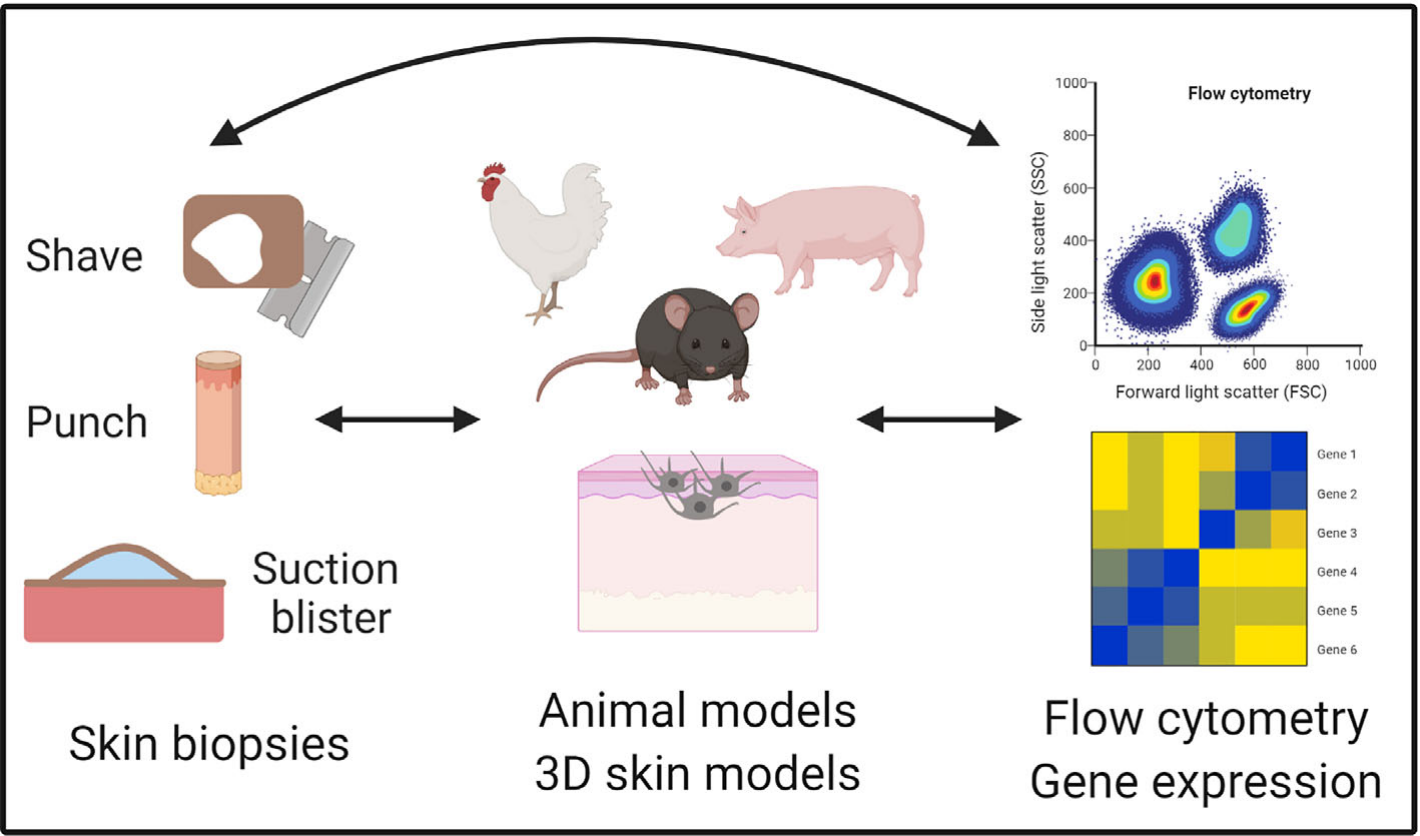
Translational tools to study vitiligo. Vitiligo is uniquely positioned for translational studies because of ready access to the skin and multiple collection techniques for human skin samples such as shave, punch, and suction blister biopsies. These biopsies can then be used for the identification and characterization of cell populations on the single cell level with technologies like flow cytometry and single cell RNA sequencing. Animal models are available for studying initiating factors and adaptive immune responses, as well as cell culture techniques like 3D skin models that have been useful for elucidating mechanisms during vitiligo pathogenesis. Figure created in BioRender.com.

Rubio et al. defined translational research as the merging of basic, clinical, and population research, which continuously build on each other (94). The multi-directionality of translational research is advantageous because it aligns mechanistic studies in a model system with clinical observations and studies on human tissue. Thus, translational research leverages the best of both basic and clinical approaches, understanding the mechanism of disease while maintaining physiologic relevance (**Figure 3**).

Vitiligo is uniquely suited to translational research because of its high prevalence in the population, opportunity for visual evaluation of disease progression, and ready access to the target tissue. Procedures for obtaining patient skin biopsies are less invasive than in other organs, such as the intestines where endoscopic procedures are required or pancreas and brain where samples can only be safely obtained postmortem. Tissue samples are critically important for understanding human disease pathogenesis, because other sources of patient tissue such as the blood is *at best* a diluted sample of disease-causing factors in the tissues, and *at worst* may be specifically depleted of these factors.

Traditional methods used to sample the skin include punch or shave biopsies, which yield small pieces of solid tissue for analysis. These biopsies can be analyzed using histology, immunohistochemistry, and immunofluorescence to determine protein expression, which enables positional information that indicates spacial relationships among cells. Tissue can be homogenized for RNA or protein quantification; however, this eliminates information on individual cells or their position within tissues. Finally, solid tissue can be enzymatically digested to yield single cells for single cell analyses such as flow cytometry, RNA sequencing, and others. Disadvantages to these approaches include pain, need for anesthesia, scarring of the sampled site, and requirement for sutures in some cases. In addition, immune cells infiltrating vitiligo lesions can be difficult to isolate, since they are frequently present in a perilesional location, somewhere beyond the lesional border. This is because these cells migrate through the skin outward from the lesion center while melanocyte loss requires epidermal turnover to observe clinically, so that the true lesion edge is located beyond the visible border. Thus, it can be very difficult to determine where the biopsy should be taken in order to “catch” migratory immune cells.

The use of suction blistering on human volunteers was first described by Kiistala and Mustakallio in 1964 for the purpose of separating the epidermis and dermis (95). Originally, suction blister roofs had been used for obtaining cells for culture, quantifying bacteria on the epidermis, and studying epidermal permeability and biochemical properties (96–98). The time blisters took to form on various skin disease states compared to healthy could also be used to categorize skin diseases and their severity, especially blistering diseases like pemphigus vulgaris (99). Blister fluid was utilized for to analyze immune cells in the skin, such as cell recruitment during allergic inflammation because an influx of immune cells into the blister occurred over multiple days after induction of the blister (100). Suction blistering later was used for epidermal grafting, using cells from both the blister roofs and fluid, to treat stable vitiligo lesions (101). More recently, this technique has been used in other contexts to identify cell populations, protein levels of soluble receptors, and secreted growth factors and cytokines (102–104).

The suction blistering approach is less invasive, not painful, does not typically scar, and does not require anesthesia or sutures. It is accomplished through application of negative pressure by a suction chamber, often with the addition of gentle heating (99). While a single punch biopsy collects 4-6 mm of skin, suction blistering can yield many more total cells because the interstitial skin fluid that forms the blister (up to 10mm) is drawn from an even larger area and each subject can contribute multiple blisters, we frequently acquire up to 10 per subject. We optimized this technique to sample the involved and uninvolved skin of subjects with vitiligo as well as healthy controls, and found that the extracted blister fluid contains multiple cell types that can be characterized by flow cytometry, as well as proteins that can be measured with ELISA (104). Thus, it is an efficient technique to isolate migratory immune cells from vitiligo lesions as well as soluble proteins from interstitial fluid. However, blisters do not preserve the architecture of the skin, and thus positional information is lost. In addition, it may preferentially sample the epidermis and upper dermis, potentially missing cells (particularly structural cells like fibroblasts and endothelial cells) and fluid from the deeper dermis (105, 106). Since vitiligo consists of melanocyte destruction at the basal epidermis, the blistering approach is well-suited for translational studies in vitiligo.

Once single cells have been obtained from skin through suction blistering or enzymatic digestion of solid tissue biopsies, several techniques can be used for analysis of the cells (**Table 1**). Flow cytometry has enabled the identification and characterization of cell phenotypes on the protein level in vitiligo patients. It does not only distinguish the number of cells that are expressing a marker of interest but can also measure the amount of protein expressed by each cell as mean fluorescence intensity (MFI). In addition, since vitiligo has known autoantigens, the use of tetramers and pentamers have been valuable tools for the detection and characterization of circulating autoreactive cells in peripheral blood and skin. Flow cytometry has limitations though, as many transcription factors and intracellular proteins are difficult to detect due to the lack of available antibodies or sufficient permeabilization methods, and some receptors are quickly internalized so protein expression by flow cytometry may not always be an accurate assessment.

**Table 1:** Table comparing the advantages and disadvantages of translational tools available to study vitiligo.

Translational tools to study vitiligo			
Technique		Advantages	Limitations
**Tissue acquision methods**	Punch Biopsy	Maintains architecture; used for histology, IHC, IF, obtaining cells for cell culture	Can cause pain; scarring of a sample site; requires anesthesia and sutures or gelfoam
	Shave Biopsy	Maintains architecture; used for histology, IHC, IF. obtaining cells for cell culture	Can cause pain; scarring of a sample site; requires anesthesia
	Suction Blister	Less invasive; not painful; non- scarring; collects more cells than conventional biopsies; provides interstitial skin fluid for analysis of soluble molecules	Samples epidermis and upper dermis; missing deeper, structural cells
**Assay methods**	Flow cytometry	Identification and characterization of single cell protein expression	Dependent on availability and quality of antibodies; limited number of proteins can be analyzed; lacks spatial information
	Histology/ Immunohistochemistry/ Immunofluorescence	Identifies immune infiltration and protein expression with spacial information is maintained	Requires optimization of staining and imaging; dependent on availability and quality of antibodies; limited number of proteins can be analyzed; less quantifiable than single-cell methods
	Assay for Transposase-Accessible Chromatin using sequencing (ATAC-seq)	Determine epigenetic changes between disease states	Epigenetic changes do not always lead to protein expression; lacks spatial information; expensive
	Single cell RNA sequencing (scRNA-seq)	Identification and characterization of single cell transcripts; does not require prior knowledge of genes of interest; no antibody optimization	Not all transcript changes reflect protein expression; lacks spatial information; expensive
	Metabolomics	Understand changes in metabolic processes between disease states	Unable to identify which cells are producing which metabolites
	Spatial mass spectrometry imaging	Identify which cell types are producing which proteins and metabolites with spatial resolution	Not as quantitative as standard metabolomics
	Enzyme-linked immunosorbent assay (ELISA)	Identify and measure soluble proteins	Does not identify which cell types produce which proteins
	Slide-seq/ RNA seqFISH+	Transcriptomics with spatial information	Fewer targets can be analyzed
	2D cell culture	Can characterize primary cells, including cell-cell interactions through co-culture	Missing physiologically relevent conditions, including 3D interactions, air-tissue interface
	3D cell culture	Modeling of 3D skin envirionment and cellular interactions; may be useful for modeling autoimmune cell-cell interactions	More complex methods; does not involve all cell interactions
	Animal models	Models in vivo disease; parallels to human disease; useful for complex cellular interactions and mechanistic studies	Costly to maintain; can be complex methods; some human pathways not present in animal models

Single cell RNA sequencing can provide valuable insights at the single cell level (107). This approach enables researchers to identify unknown immune cell interactions through signaling networks with ligand/receptor pairing, unlike flow cytometry, which detects a small set of previously selected target proteins. It also can use gene expression within individual cells to discover new cell subtypes, which is not possible through bulk tissue gene expression analysis. However, not all changes on the RNA transcript level are reflected at the protein level. Further regulation can occur post-transcriptionally and post-translationally, which can affect whether a protein is available or functional. Therefore, validation studies must be done to determine whether RNA expression changes are reflected at the protein level.

Assay for Transposase-Accessible Chromatin using sequencing (ATAC-seq) is a technique to assess genome-wide chromatin accessibility by probing open chromatin with hyperactive transposases that are pre-loaded with sequencing adaptors (108). This technique can be performed in bulk sequencing and on single cells (109). If done at the single cell level, cell specific transcription factors, binding sites and changes in cell-to-cell DNA accessibility variability within different disease states can be explored (110). These epigenetic approaches could be applied to understanding vitiligo pathogenesis, as already described for Systemic Lupus Erythematosus (SLE) (111). However, open chromatin does not always lead to higher expression of genes or their expression as proteins, and thus further studies are required to validate findings at this level, similar to RNA sequencing.

Interstitial fluid, which cannot be obtained using conventional biopsy approaches, can be used to analyze soluble factors that might contribute to disease pathogenesis. For example, we used ELISA to determine that CXCL9 was a sensitive and specific biomarker of disease activity in vitiligo lesions (104). Metabolomics has been performed on interstitial skin fluid, which could be used to understand changes in metabolic processes within nonlesional and lesional skin (112). While soluble factors in fluid cannot be measured at the single cell level, when combined with single cell techniques like scRNA-seq, pathways can be inferred, and new hypotheses developed. These tools will provide new insight into vitiligo pathogenesis and potentially fast track the discovery of new pathways and mechanisms involved in vitiligo, in hopes of developing better and durable therapeutic options. All these techniques would provide a wealth of new and exciting insights into vitiligo pathogenesis. While current techniques using suction blister fluid lack spatial information, emerging techniques seek to examine this, including spatial transcriptomics like Slide-seq and RNA seqFISH+, as well as spatial mass spectrometry imaging (113, 114).

All biopsy techniques allow for the isolation of cells for culture. While healthy melanocytes can be cultured, melanocytes from vitiligo lesions were difficult to establish *in vitro* because they had a long lag time for growth and could not be passaged (42, 115). In the early 1990’s, cell culture techniques improved to enable the culture of both healthy and vitiligo melanocytes by the addition of growth factors, such as fibroblast growth factor (bFGF), phorbol 12-myristate 13-acetate (PMA), and bovine pituitary extract (BPE) (115, 116). This has supported investigation of melanocyte biology and autoimmune killing in co-culture experiments. There are limitations to what can studied in 2D cell culture however, and so the future development of re-creating the skin environment *in vitro* with 3D skin cultures may help further dissect mechanisms of autoimmunity from more physiologically relevant interactions (117–119). This has been used by Van den Boorn et al., who cultured T cells isolated from punch biopsies with additional biopsies from normal skin from the same patient (62). Boukhedouni et al. also used 3D skin model to study the effect of TNFα on melanocyte adhesions (56).

Translational research covers a vast range of experimental designs. While many only consider experiments containing human samples and clinical studies to be translational, others consider animal models that parallel human disease to be translational work as well. It is easy to recognize the direct impact for patients when working with patient samples, but these studies tend to lack mechanistic insights in this context, which basic research is better positioned to address. Therefore, the development and use of animal models has helped to add mechanistic understanding to clinical observations. Animal models of vitiligo have been extensively reviewed by Essien and Harris (120). Models of vitiligo include both spontaneous and induced systems, and spontaneous models include Smyth line chickens, Sinclair swine, Gray-allele horses, and a variety of mouse models (121–125). These are useful for studying disease initiation but can be costly to maintain and may be more difficult to study because of limited reagents available that are compatible with these species. Inducible models are helpful to study disease progression after onset and include the mouse model developed by Harris et al. that parallels human disease by developing patches of skin depigmentation without involving hair, and thus has the potential to repigment with treatment. Like humans, this model is mediated by CD8+ T cells and exhibits IFN-γ driven disease with a similar Type 1 cytokine signature (126–128). Animal models are most useful for focused studies that probe specific questions for which the model is optimized to answer. For example, questions about disease initiation, including genetic influences, should be studied in spontaneous models, models designed for this purpose, or directly in humans, since many induced models bypass this step of pathogenesis.

## Lessons Learned from Translational Research

**Figure 4:**
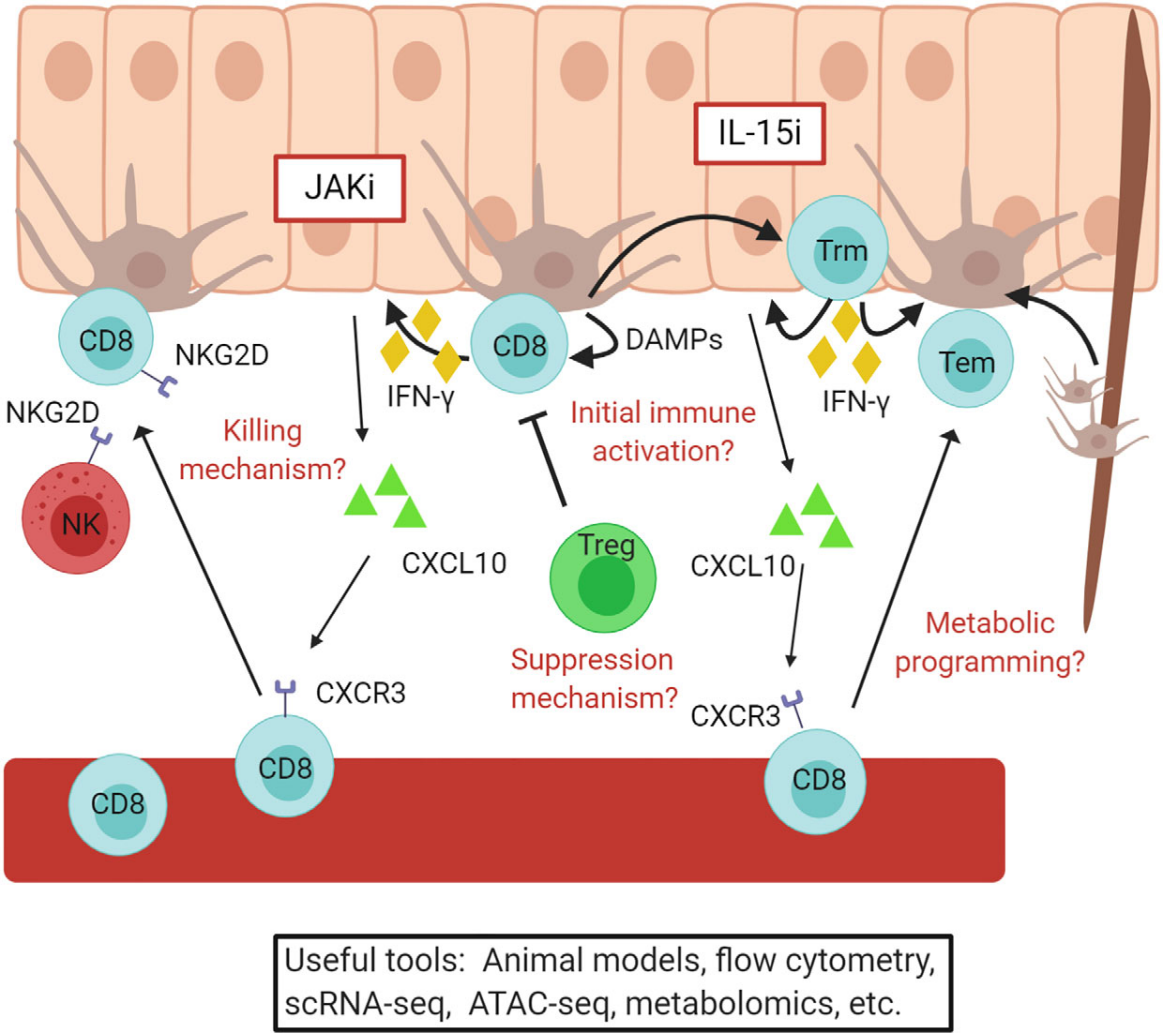
Vitiligo pathogenesis revealed through translational research. A simple overview of the current understanding of vitiligo pathogenesis. Autoreactive CD8+ T cells are recruited to the skin by CXCL10 produced by keratinocytes and kill melanocytes. These cells convert to resident memory T cells, which maintain vitiligo and require IL-15 signaling in the skin. JAKi and IL15i, outlined in red, may be effective treatments for vitiligo and are currently being tested in clinical trials. Some questions that remain include how melanocyte abnormalities initiate autoimmunity, the mechanism by which CD8+ T cells kill melanocytes, how Tregs suppress disease, and how changes in metabolism effect disease activity. Existing and developing translational tools will help to answer these and other questions. Figure created in BioRender.com.

The long history of studying vitiligo using translational methods has enabled us to develop a more complete picture about disease pathogenesis, which has been more difficult to achieve in diseases that target less-accessible tissues. Translational studies have been great assets for understanding the mechanisms of disease, which can then promote the development of promising new treatments (**Figure 4**). For instance, patient biopsies revealed that there is an influx of melanocyte specific CD8+ T cells into lesional skin, which produce IFNγ and IFNγ-induced genes, and express NKG2D (39, 62, 127, 129–131). One study reported type I IFN expression within lesions using CXCL9 and MxA as surrogate markers induced by IFNs, however these can be induced by IFNγ as well. Mouse studies do not support a role for type I IFNs in vitiligo, as IFNα receptor (IFNaR) knockout mice still develop vitiligo to the same extent (132). Thus, while IFNγ clearly plays an important role in vitiligo pathogenesis, it is still unclear whether other IFNs contribute to disease.

Infiltrating CD8+ T cells express CXCR3 at high levels within lesions, and expression of CXCR3 ligands are characteristic of vitiligo lesions (104, 127, 133, 134). Supporting data using mouse models reveal that CXCR3 and CXCR3 ligands are expressed within lesional vitiligo skin in mouse models, and it is required for recruitment of T cells during disease progression and maintenance (127, 135, 136). Mouse models also made it possible to determine that keratinocytes respond to IFNγ production from T cells and secrete CXCL10 to recruit melanocyte-reactive CD8+ T cells to the skin (55). These discoveries provided the rationale to test JAK inhibitors, which block IFNγ signaling, as treatments for vitiligo and eventually led to a successful Phase 2 clinical trial to test topical ruxolitinib as a novel treatment for the disease (137–143).

However, conventional treatments (144, 145) and even JAK inhibitors (137, 139) do not appear to provide durable responses following treatment, as disease relapses within previous lesions once they are stopped. Translational studies revealed that autoreactive CD8+ T cells form tissue resident memory cells (Trm) in vitiligo lesional skin (133, 146, 147). Mouse models confirmed the presence of Trm in vitiligo lesions and revealed that they are responsible for maintenance and relapse of disease after stopping treatment (146, 148–150). Additional studies determined that melanocyte-specific autoreactive Trm require IL-15, and that targeting IL-15 signaling eliminates Trm from the skin and results in durable repigmentation in mice (146). IL-15 expression is elevated in vitiligo lesional skin and may be induced by oxidative stress in keratinocytes (151). Therefore, targeting IL-15 may provide a durable treatment option for vitiligo, and clinical trials to test this hypothesis are imminent (152).

As mentioned above, increased expression of HSP70i has been reported in vitiligo lesions, and activates skin dendritic cells that could prime autoreactive T cells against melanocytes (89). A mouse model required HSP70i for vitiligo initiation and when inhibited, disease is prevented (66, 89). Additionally, melanocytes produce HSP70 in response to oxidative stress, which may provide a link between cellular stress in melanocytes and inflammation that initiates autoimmunity in vitiligo (92, 153). Therefore, melanocyte stress could be mitigated by targeting this pathway and thus provide a new opportunity for treatment.

Regazzetti et al. reported a decreased expression of WNT signaling components in melanocytes when exposed to oxidative stress. The authors went on to demonstrate that treatment with a chemical WNT agonist can promote melanoblast differentiation in vitiligo skin, suggesting that activating WNT signaling in melanocytes might promote their regeneration (154). While that study did not show whether this differentiation fully progresses to a mature functional melanocyte, Yamada et al. reported that WNT signaling is critical in melanocyte stem cell differentiation and that WNTs are secreted predominantly by keratinocytes and melanocytes (154, 155). Furthermore, WNT signaling regulates E-cadherin, an important adhesion molecule for melanocytes, through the availability of β-catenin (156). Wagner et al. reported that there is a reduction of E-cadherin expression on melanocytes in vitiligo skin (51). Together these studies suggest that a decrease in WNT signaling might impair melanocyte adhesion. Additionally, Boukhedouni *et al*. reported that the type 1 cytokines IFNγ and TNFα impair melanocyte adhesion from the epidermis by inhibiting melanocyte E-cadherin expression and inducing keratinocyte release of protease MMP-9 (56). MMP-9 cleaves E-cadherin complexes, which results in melanocyte detachment from the epidermis. This study also reported that when type 1 cytokines were inhibited with JAK inhibitors, or when MMP-9 activity was inhibited, fewer melanocytes detached (56). Therefore, anti-inflammatory treatments like JAK inhibitors may also stabilize melanocytes and potentially could become more durable if used in conjunction with WNT agonists or inhibitors of detachment to promote melanocyte regeneration and maintenance.

Some have suggested that activating immune checkpoint inhibitors, which are receptors expressed on the surface of T cells that negatively regulate their activity, might be an effective therapeutic target for vitiligo (157, 158). Blocking cutaneous lymphocyte antigen-4 (CTLA-4) and program cell death protein 1 (PD-1) for melanoma treatment induce and exacerbate vitiligo, presumably by removing negative regulation that prevents autoimmune activation of these cells (159–162). Decreased CTLA-4 mRNA expression has been reported in whole blood of vitiligo patients, and PD-1 expression on CD8+ T cells is positively correlated with disease activity, suggesting increasing activation (163, 164). PD-L1 inhibits cytokine production and is associated with maintaining peripheral tolerance (165, 166). Miao et al. reported that the injection of a PD-L1 fusion protein into a mouse model of vitiligo led to reduced disease severity and an increased Treg to effector T cell ratio (167). However, PD-1 is also a marker for T cell exhaustion and its upregulation was reported on regulatory T cells in the peripheral blood of vitiligo patients (168). Theoretically, PD-L1 may negatively regulate Tregs similarly to effector T cells, however neutralization of PD-L1 *in vitro* led to an expansion of human Tregs isolated from chronically infected HCV patients (169). Targeting these or other negative stimulatory molecules may limit the autoreactive CD8+ T cell activity, which may allow for Tregs to recover to reinstate tolerance, but further investigation is needed.

There have been recent advancements in Treg-based therapeutics. Tregs appear to actively prevent vitiligo in healthy subjects and may be impaired in those with the disease (170). Many report possible defects in Tregs in vitiligo patients, although they do not necessarily agree whether the defects are in total Treg number, ability to migrate to the skin, or suppressive function (168, 171–176). Expansion of Tregs and adoptive transfer of these cells into patients have promising results in mitigating some autoimmune diseases and transplantation (177, 178). Selective expansion of antigen-specific Tregs reportedly controls inflammation in mouse studies to a greater extent than administration of polyclonal Tregs (179–181). Chimeric antigen receptor (CAR)-Tregs are being developed as treatment options for autoimmunity and GVHD. CAR-Tregs allow for a more stable, antigen-specific Treg population that can be engineered to migrate to the site of inflammation, however more improvements are needed as off-target immunosuppression can result in susceptibility to infections and cancer development (177, 182, 183). Chatterjee et al. reported that adoptively transferred Tregs into a spontaneous vitiligo mouse model reduced disease onset. In addition, treating these mice with rapamycin limited disease progression and increased the number of circulating Tregs (184). Further investigation will be important to optimize these therapies and reduce risks for the treatment of vitiligo.

## Using Vitiligo to Understand Organ-Specific Autoimmunity

Progress made through translational research in vitiligo may facilitate progress in other diseases that are more difficult to study because they are less common and/or because target tissues are less accessible for translational research. Diseases in which CD8+ T cells target a single cell type resulting in loss of that cell type likely share a similar pathogenesis with vitiligo (92). Examples include type 1 diabetes (T1D) within the pancreas, Hashimoto’s thyroiditis in the thyroid, Addison’s disease in the adrenal cortex, and multiple sclerosis (MS) in the brain. In support of this hypothesis, GWA studies reveal common risk SNPs (92, 185–188). In addition, studies have implicated CD8+ T cells in Hashimoto’s thyroiditis, MS, and Addison’s Disease, similar to vitiligo (189–192), as well as an important role for the IFN-γ chemokine axis during pathogenesis. IFN-γ and CXCL10 are expressed in human islets to recruit lymphocytes in T1D, autoreactive cells produce IFN-γ in Addison’s Disease, and IFN-γ level positively correlates with disease severity in Hashimoto’s thyroiditis (193–196). IFN-γ is also involved in MS, although its role is still not fully understood (197). Furthermore, targeting IL-15 in a NOD mouse model reduced disease severity, suggesting IL-15 may play a role in T1D pathogenesis (198). Hence, vitiligo pathogenesis can be used as a guide for these diseases to launch more targeted investigations, as well as repurposing treatments. Narrowing the potential areas for investigation can help accelerate the understanding of pathogenesis for these autoimmune diseases.

## Conclusion

Vitiligo is an organ-specific autoimmune disease that results in white patchy skin depigmentation. Since the beginning of modern vitiligo research over 40 years ago, translational work has been at the forefront of vitiligo studies because of the simple, non-invasive collection of patient skin samples. These observations have led to clinical studies and a successful clinical trial that are likely to change how we manage patients in the clinic. In addition to sample collection, the discussion, collaboration, and open-minded consideration among researchers have made vitiligo one of the best understood autoimmune diseases. Independent, parallel studies addressing both the degenerative and autoimmune theories of pathogenesis have led to a broader understanding of vitiligo pathogenesis by preventing narrow of experimental focus. This has not only helped our understanding of vitiligo but has also provided a valuable platform to study organ-specific autoimmunity in general. The knowledge gained about vitiligo may be beneficial for those who study similar autoimmune diseases that do not have ready access to tissues for translational studies. Our understanding for vitiligo and other autoimmune disease will improve as more discussions, collaborations, and technology continue to advance. These advancements will be continuously applied toward new therapeutic development to help patients.

## Author Contributions

Wrote the paper: EK and JEH. All authors contributed to the article and approved the submitted version.

## Funding

“NIH R33/R61 AR073042”, “NIH 5 R01 AR069114”, “Hartford Foundation”, and “Vitiligo Research Fund supported by many gifts of all sizes”.

## Conflict of Interest

JEH is a consultant for Pfizer, Genzyme/Sanofi, Aclaris Therapeutics Inc, Incyte, Rheos Medicines, Sun Pharmaceuticals, LEO Pharma, Villaris Therapeutics Inc, Dermavant, Temprian, AbbVie Inc, Janssen, TeVido BioDevices, EMD Serono, Almirall, Boston Pharma, Sonoma Biotherapeutics Inc, Methuselah Health, Twi Biotech, Pandion, Cogen Therapeutics Inc, Admirx, BridgeBio, AnaptysBio, Avita, and Frazier Management. JEH is an investigator for Pfizer, Genzyme/Sanofi, Aclaris Therapeutics Inc, Incyte, Rheos Medicines, Sun Pharmaceuticals, LEO Pharma, Villaris Therapeutics Inc, Dermavant, AbbVie, TeVido BioDevices, EMD Serono, and Pandion. JEH is scientific founder of Villaris Therapeutics, Inc, which develops therapeutic treatments for vitiligo, and NIRA Biosciences. JEH is an inventor on patent #62489191 “Diagnosis and Treatment of Vitiligo” that includes targeting IL-15 and Trm for treatment of vitiligo, as well as #067988 “Anti-Human CXCR3 Antibodies for Treatment of Vitiligo” and #029531 “Compositions and Methods for Treating Vitiligo”.

The remaining authors declare that the research was conducted in the absence of any commercial or financial relationships that could be construed as a potential conflict of interest.

## Supplementary Material

Supplementary Table 1 Table describing key papers showcasing the inconsistencies within the neuronal hypothesis. Green indicates studies supporting the neuronal hypothesis, while red indicates studies not supporting the neuronal hypothesis.
